# Editorial: Obesity and chronic kidney disease: complexities, clinical impact, and challenges in nutritional management

**DOI:** 10.3389/fnut.2023.1212700

**Published:** 2023-08-01

**Authors:** Caterina Conte, Alessio Molfino

**Affiliations:** ^1^Department of Human Sciences and Promotion of the Quality of Life, San Raffaele Roma Open University, Rome, Italy; ^2^Department of Endocrinology, Nutrition and Metabolic Diseases, IRCCS MultiMedica, Milan, Italy; ^3^Department of Translational and Precision Medicine, Sapienza University of Rome, Rome, Italy

**Keywords:** obesity, chronic kidney disease, nutrition, obesity paradox, adiposity

Obesity and chronic kidney disease (CKD) are becoming increasingly common and have reached epidemic levels (1, 2). Furthermore, obesity is a risk factor for CKD and may hasten its progression (3). In patients with end-stage renal disease (ESRD), obesity may prevent or delay access to a kidney transplant as well as affect peri- and post-transplantation outcomes (4, 5). However, an “obesity paradox,” or a protective association between obesity and survival in ESRD patients, particularly in older patients on haemodialysis, has been suggested by observational studies (6). This raises some doubt as to whether all patients with CKD and obesity would benefit from intentional weight loss.

Depending on the stage of CKD and due to the dietary restrictions imposed by electrolyte and acid-base imbalances, water and salt retention, and metabolic changes linked to CKD, the nutritional management of patients with CKD is challenging, especially when obesity is also present. To make things more complex, there are many unexplored issues and unmet needs in renal nutrition. Thus, the Research Topic “*Obesity and chronic kidney disease: complexities, clinical impact, and challenges in nutritional management*” was launched to increase awareness of the impact that obesity has on kidney outcomes, and to provide evidence-based information useful to improve the nutritional management of patients with obesity and CKD.

Studies included in this Research Topic explored the role of diet and specific nutrients on morbidity and mortality in CKD, as well as on renal outcomes. Huang et al. examined the relationship between the energy-adjusted dietary inflammatory index (E-DII) and 5-year mortality in more than 7,200 individuals participating in the National Health and Nutrition Examination Surveys (NHANES). They showed that a pro-inflammatory diet, as indicated by a higher E-DII significantly associates with 5-year all cause and cardiovascular mortality in the general population, and that this association is stronger in patients with reduced kidney function (CKD 3-5) as compared with individuals with normal function. Zhang et al. examined the association between dietary selenium and microalbuminuria in more than 8,500 individuals with obesity participating in the NHANES. In CKD, selenium and the selenium-dependent antioxidant enzyme glutathione peroxidase are often reduced, which may associate with increased generation of reactive oxygen species (7). However, excess selenium intake may have detrimental effects on the kidney (8). Zhang et al. found that excess selenium intake was significantly associated with a higher risk of developing microalbuminuria in females with obesity, but not in males with obesity. The studies by Huang et al. and Zhang et al. highlight the highly influential role of diet and specific nutrients in the context of CKD, and corroborate the concept of “food as medicine” that has gained popularity in recent years (9).

Chen et al. investigated the association of obesity-related parameters with CKD in middle-aged and elderly (aged ≥ 50 years) Taiwanese individuals with or without CKD (as defined by an estimated glomerular filtration rate [eGFR] < 60 ml/min/1.73 m^2^ or urinary albumin to creatinine ratio ≥ 30). They focused on the ratio between triglycerides and high-density lipoprotein-cholesterol (TG/HDL-C), a novel biomarker for predicting the risk of metabolic syndrome and cardiovascular disease (10). They found a significant association between the TG/HDL-C ratio and CKD, estimated glomerular filtration rate (eGFR), as well as cardiovascular risk factors. Although the cross-sectional nature of this study cannot establish causation, these results highlight the strict connection between CKD and metabolic alterations. In fact, both quantitative and qualitative lipid alterations are observed in patients with CKD, which contribute to the increased cardiovascular risk in this population (11).

Whether the obesity paradox is a real entity is still matter of debate (12). It should be emphasized that most studies used BMI to define obesity. However, the BMI only provides limited information, and does not inform on adiposity and adipose tissue distribution.

To shed light on this issue, Shen et al. analyzed data from 3,262 Asian patients with stage 3–5 CKD according to percent total body fat (TBF%) and waist-to-height ratio (WHtR) quartiles. Their analysis confirmed a survival advantage with increasing BMI, whereas an increase in all-cause mortality was evident for individuals in the highest TBF% or WHtR quartile in the whole cohort. Furthermore, individuals with normal weight (BMI < 25 kg/m^2^) or normal WHtR but in the highest TBF% quartile had a 3.8- and 2.9-fold increase in the risk of all-cause mortality as compared with individuals in the lowest TBF% quartile. These data point at TBF% as an interesting biomarker to identify individuals with normal weight and/or normal waist circumference who are at increased risk of mortality and confirm that BMI might not be the most reliable marker of body composition, challenging once again the “obesity paradox.”

In conclusion, the nutritional management of patients with CKD should take into account several aspects, from an adequate balance of dietary components (Zhang et al.) to the proinflammatory potential of the diet (Huang et al.) (Figure 1). A global assessment of patients with CKD should be performed, taking into account the lipid profile to tackle the cardiovascular risk (Chen et al.), and the anthropometric evaluation should not be limited to BMI, as even individuals with normal weight might be at increased risk of mortality (Shen et al.). In this light, the importance of assessing body composition is being increasingly recognized. On one hand, these relatively simple strategies should become routinary in clinical practice. On the other hand, further studies are needed to refine the nutritional management of patients with CKD.

**Figure 1:**
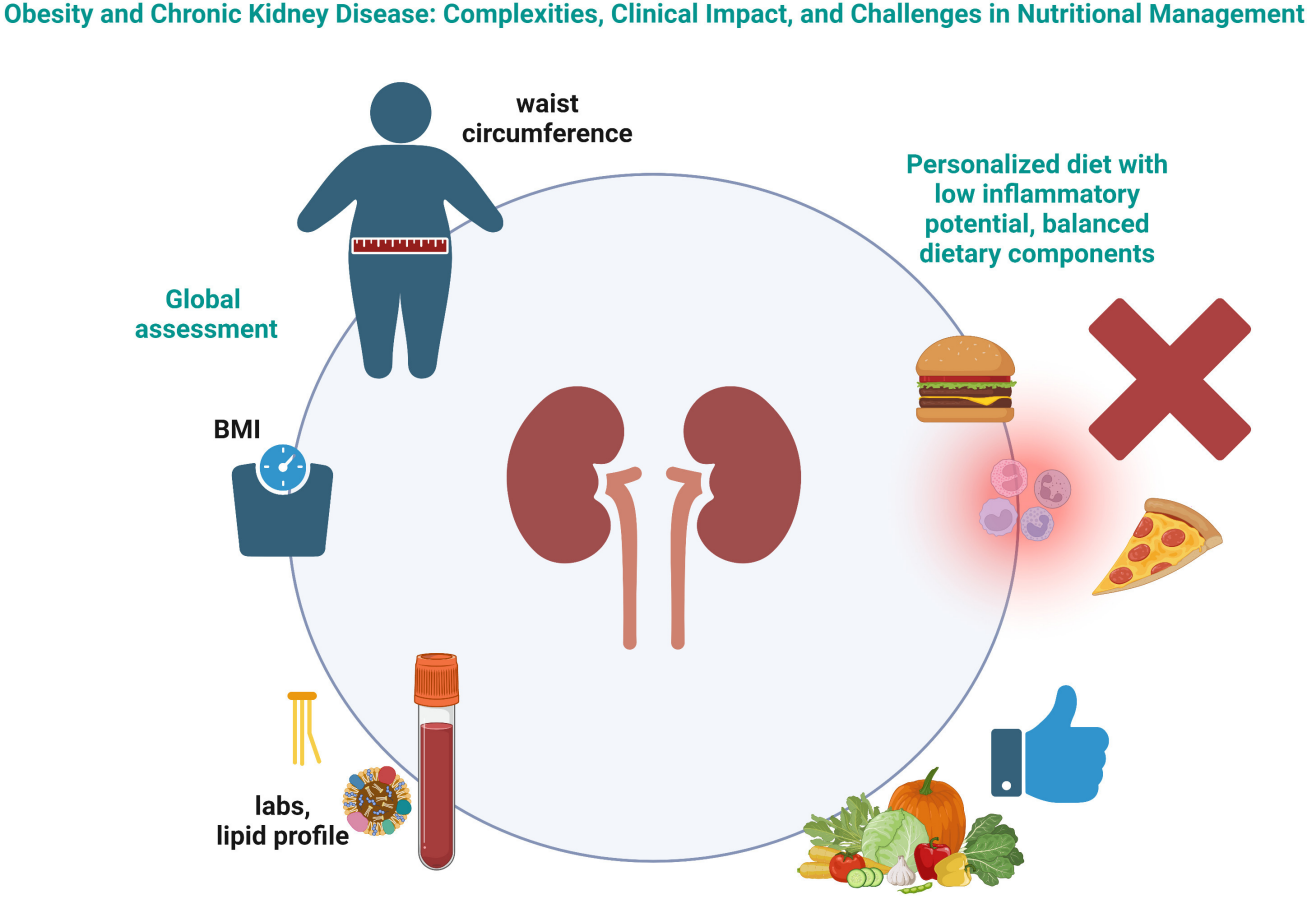
Schematic representation of the complexities in the nutritional management of patients with chronic kidney disease. Created with BioRender.com.

## Author contributions

CC and AM contributed to the conception of the work, drafted the work and revised it critically for important intellectual content, provided approval for publication of the content, and agree to be accountable for all aspects of the work in ensuring that questions related to the accuracy or integrity of any part of the work are appropriately investigated and resolved. All authors contributed to the article and approved the submitted version.

## References

[1] Janssen F Bardoutsos A Vidra N. Obesity prevalence in the long-term future in 18 European Countries and in the USA. Obes Facts. (2020) 13:514–27. 10.1159/000511023 33075798 PMC7670332

[2] Sundström J Bodegard J Bollmann A Vervloet MG Mark PB Karasik A. Prevalence, outcomes, and cost of chronic kidney disease in a contemporary population of 2.4 million patients from 11 countries: The CaReMe CKD study. Lancet Reg Health Eur. (2022) 20:100438. 10.1016/j.lanepe.2022.100438 36090671 PMC9459126

[3] Nawaz S Chinnadurai R Al-Chalabi S Evans P Kalra PA Syed AA. Obesity and chronic kidney disease: A current review. Obes Sci Pract. (2023) 9:61–74. 10.1002/osp4.629 37034567 PMC10073820

[4] Diwan TS Cuffy MC Linares-Cervantes I Govil A. Impact of obesity on dialysis and transplant and its management. Semin Dial. (2020) 33:279–85. 10.1111/sdi.12876 32277512

[5] Glicklich D Mustafa MR. Obesity in kidney transplantation: impact on transplant candidates, recipients, and donors. Cardiol Rev. (2019) 27:63–72. 10.1097/CRD.0000000000000216 29870421

[6] Ziolkowski SL Long J Baker JF Chertow GM Leonard MB. Chronic kidney disease and the adiposity paradox: valid or confounded? J Ren Nutr. (2019) 29:521–8. 10.1053/j.jrn.2018.11.011 30709713 PMC6663655

[7] Stockler-Pinto MB Mafra D Moraes C Lobo J Boaventura GT Farage NE. Brazil nut (Bertholletia excelsa, HBK) improves oxidative stress and inflammation biomarkers in hemodialysis patients. Biol Trace Elem Res. (2014) 158:105–12. 10.1007/s12011-014-9904-z 24504745

[8] Vinceti M Filippini T Cilloni S Bargellini A Vergoni AV Tsatsakis A. Health risk assessment of environmental selenium: Emerging evidence and challenges (Review). Mol Med Rep. (2017) 15:3323–35. 10.3892/mmr.2017.6377 28339083 PMC5428396

[9] Mafra D Borges NA Lindholm B Shiels PG Evenepoel P Stenvinkel P. Food as medicine: targeting the uraemic phenotype in chronic kidney disease. Nat Rev Nephrol. (2021) 17:153–71. 10.1038/s41581-020-00345-8 32963366

[10] Kosmas CE Rodriguez Polanco S Bousvarou MD Papakonstantinou EJ Pena Genao E Guzman E. The triglyceride/high-density lipoprotein cholesterol (TG/HDL-C) ratio as a risk marker for metabolic syndrome and cardiovascular disease. Diagnostics (Basel). (2023) 13:929. 10.3390/diagnostics13050929 36900073 PMC10001260

[11] Noels H Lehrke M Vanholder R Jankowski J. Lipoproteins and fatty acids in chronic kidney disease: molecular and metabolic alterations. Nat Rev Nephrol. (2021) 17:528–42. 10.1038/s41581-021-00423-5 33972752

[12] Simati S Kokkinos A Dalamaga M Argyrakopoulou G. Obesity paradox: fact or fiction? Curr Obes Rep. (2023) 12:75–85. 10.1007/s13679-023-00497-1 36808566

